# *Tessaria absinthioides* (Hook. & Arn.) DC. Determines Inhibition of Tumor Growth and Metastasis *In Vitro* and *In Vivo* in Murine Melanoma

**DOI:** 10.3390/plants14091379

**Published:** 2025-05-02

**Authors:** Lourdes Inés Pascual, Sebastián Real, Arianna Sosa-Lochedino, Fiorella Campo Verde Arbocco, María Belén Hapon, Carlos Gamarra-Luques

**Affiliations:** 1 Instituto de Medicina y Biología Experimental de Cuyo (IMBECU), Universidad Nacional de Cuyo, CCT Mendoza CONICET, Ruiz Leal s/n, Mendoza 5500, Provincia de Mendoza, Argentina; lourdes.p.97@gmail.com (L.I.P.); arboccocv@gmail.com (F.C.V.A.); bhapon@mendoza-conicet.gob.ar (M.B.H.); 2 Instituto de Histología y Embriología de Cuyo (IHEM), Universidad Nacional de Cuyo, CCT Mendoza CONICET, Centro Universitario, Ciudad de Mendoza M5502JJMA, Mendoza, Argentina; tazreal@gmail.com; 3 Instituto de Fisiología, Facultad de Ciencias Médicas (FCM), Universidad Nacional de Cuyo , Centro Universitario, Ciudad de Mendoza M5502JJMA, Mendoza, Argentina; 4 Instituto de Investigaciones en Tecnologías Energéticas y Materiales Avanzados (IITEMA), Universidad Nacional de Río Cuarto, Ruta 36 Km 601, Río Cuarto CP5800, Córdoba, Argentina; ariannasosalochedino@gmail.com; 5 Facultad de Ciencias Exactas y Naturales, Universidad Nacional de Cuyo , Padre Jorge Contreras 1300, Parque General San Martín, Mendoza M5502JMA, Provincia de Mendoza, Argentina

**Keywords:** botanical compounds, *Asteraceae*, cytotoxicity, antitumoral, antimetastatic

## Abstract

Melanoma is one of the deathliest cancers worldwide and its incidence is reaching epidemic proportions. It is characterized by intrinsic chemo-resistance, low response rates to treatment and high metastatic potential. Because of this, new therapeutic options are permanently required. *Tessaria absinthioides* (Hook. & Arn.) DC. is a traditional medicinal plant, with antioxidant, selective cytotoxicity and anti-colorectal cancer evidence-based properties. This study aims to demonstrate the antitumoral and antimetastatic effects of *T. absinthioides* decoction (DETa), correlating *in vitro* and *in vivo* activities in a murine melanoma model. DETa was assayed on B16F0 murine non-metastatic cells to determine cytotoxicity and clonogenicity; while, in the B16F10 metastatic siblings, adhesion, wound healing migration and Boyden chamber invasion were studied. The *ex vivo* intestinal-sac model was used to quantify DETa bioavailability. Meanwhile, in C57BL6/wt mice, DETa was orally administered to evaluate its antitumoral and antimetastatic activities. DETa induced cytotoxicity in a dose- and time-dependent manner, affecting the long-term clonogenic survival, as well as the processes of adhesion and migration. Then, the intestinal absorption of DETa phenolics was proven, while the systemic anti-tumoral and anti-metastatic activities of DETa were confirmed. Results demonstrated that DETa has antimelanoma activity promoting this botanical compound as a relevant agent for cancer research and treatment.

## 1. Introduction

Melanoma is a malignant form of cancer derived from melanocyte cells. Arising in 90% of cases, cutaneous melanoma is the most common disease presentation. Although the white population is mainly affected, acral melanomas are most frequent among populations with black or brown skin color and East Asian people [1]. While the incidence and mortality of most cancers have declined over the past decades, the incidence of melanoma has dramatically increased, reaching epidemic proportions. Globally, 325,000 new cases were diagnosed in 2020; about 1% of these cases will become invasive, being responsible for 75% of skin cancer deaths [2]. The situation got worse due to the COVID-19 pandemic, where malignant melanoma demonstrated diminished screening, diagnosis and treatment, resulting in a high number of patients with advanced disease stages [3]. Currently, the most common clinical treatment of melanoma is surgical excision, followed by radiation, immunotherapy or chemotherapy. Despite this, owing to the intrinsic chemo-resistance of melanoma cells, the treatment has low response rates and presents severe side effects [4]. For the aforementioned reasons, the development of new alternatives with high activity and low toxicity for treating melanoma is critical.

There is a continuous effort to improve the treatment outcomes of melanoma. Among the promising alternatives, natural plant-derived compounds are a notable source of antitumor agents due to their lower toxicity and increased bioavailability. Numerous studies attribute the effects of plants on melanoma to polyphenols, highlighting their potential activity as antioxidant and anti-proliferative agents, which act on different signaling pathways, supporting their value as anticancer compounds [5]. In the field of complementary and alternative medicine therapies, botanical agents, phytochemicals, herbal extracts and formulas are frequently used by skin cancer patients—specifically, in melanoma. This use currently reaches up to 40–50% of diagnosed patients [6].

Recently, An et al. [7] systematically reviewed 52 studies that evaluate the anti-melanoma effects of natural products originally reported by traditional medicine. Among the most recognized examples of plant extract contributions, it is appropriate to mention the case of parsley (*Petroselinum crispum*) leaf extract, where the activity against melanoma cells has been attributed to its flavone content, evidencing antioxidative and antiproliferative activity [8]. Furthermore, *Pinus maritima* extract has been reported to affect A375 cells due to its procyanidin content, by reducing reactive oxygen species formation, inducing apoptosis through increased expression of cleaved caspase-3 and suppressing cellular invasion via downregulation of matrix metalloproteinase-9 [9]. In addition, the aqueous extract of *Melissa officinalis*, which contains 18 different polyphenols, has been reported as an antiangiogenic botanical compound that exerts selective cytotoxicity against A375 human melanoma cells [10]. Finally, it is relevant to mention the activity of the *Prosopis strombulifera* aqueous extract, which contains 26 phenolic compounds and is able to induce G_2_/M cell cycle arrest and apoptosis on B16F0 melanoma cells by increasing the expressions of p21^cip1^, cleaved caspase-3 and cleaved PARP [11].

*T. absinthioides* (Hook. & Arn.) DC, from the Asteraceae family, popularly known as “pájaro bobo”, is an aromatic plant traditionally used by the Aymara and Huarpe native populations throughout the Argentinian and Chilean territories. Its aqueous preparations were used as a balsamic, expectorant and hypocholesterolemic agent, and they have been employed in cases of hepatitis, renal insufficiency, diabetes and digestive disorders [12]. Recent evidence-based scientific studies focused on decoctions of *T. absinthioides* (DETa) have described its chemical composition and several biological properties. DETa characterization, determined by ultrahigh resolution liquid chromatography orbitrap MS analysis (UHPLC-PDA-OT-MS), revealed 30 different phenolic compounds, including sesquiterpenes, flavonoids and phenolic acids [13]. Recently, in a study on different annual harvests of *T. absinthioides*, our group reported DETa’s bioactive and phytochemical markers, identifying five compounds as tumoral cytotoxic markers: Apigenin and Chlorogenic, *p*-Coumaric, Tessaric and Vanillic acids[14]. Through studies on laboratory animals, the oral acute, subacute and chronic toxicity of DETa were discarded. Also, the beneficial effects induced by its oral administration, such as reduction in total cholesterol, triglycerides and glucose levels were described. Additionally, antioxidant effects and attenuated atherogenesis were also reported [15,16,17]. Specifically, in relation to cancer, DETa demonstrates selective cytotoxicity towards cancer cells, affecting glioblastoma, cervicouterine, mammary and colorectal cancer cells more than epithelial non-tumoral cell lines. Moreover, in a murine model of induced colorectal cancer, the oral administration of DETa at a dose of 300 mg/kg/day significantly increased overall survival compared to both untreated control and chemotherapy-treated animals [15].

In light of the aforementioned precedents, the aim of this study is to demonstrate the antitumoral and antimetastatic effects of DETa by correlating *in vitro* and *in vivo* activities in a murine melanoma model to promote further research and its use as an anticancer botanical.

## 2. Results

### 2.1. In Vitro Study of DETa Cytotoxicity on Melanoma Non-Metastatic Cell Line

To demonstrate the capability of DETa to induce cytotoxic effects on non-metastatic melanoma cells (B16F0), dose-response and time-course experimental designs were used.

**Figure 1:**
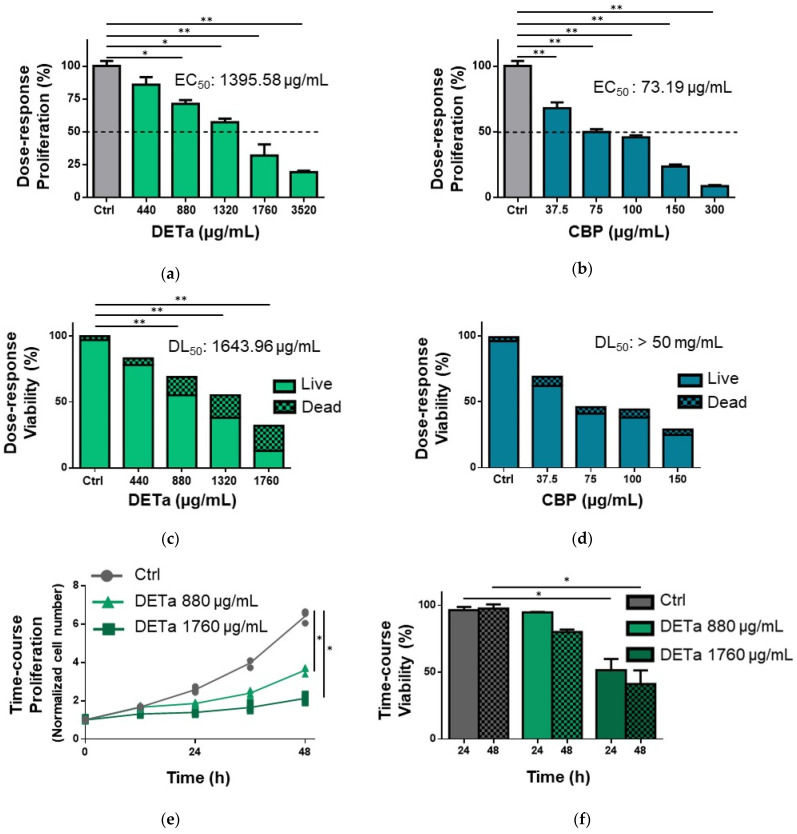
DETa induces cytotoxicity on B16F0 melanoma cells in a dose- and time-dependent manner. Panels (**a**,**b**) show a dose-response effect of DETa and CBP on cell proliferation studied by the MTT assay after 48 h of treatment. Panels (**c**,**d**) present the changes in cell viability induced by DETa and CBP analyzed by the dye exclusion assay at 48 h. In panel (**e**) are the time-course changes in cell proliferation at 12, 24, 36 and 48 h. Panel (**f**) displays the time-course reduction of cell viability. EC_50_: Concentration of treatment needed to inhibit 50% proliferation. DL_50_: Treatment concentration where 50% of cell death was observed. Ctrl: control untreated group. DETa: *T. absinthioides* decoction. CBP: Carboplatin. *: Indicates significant differences between groups (ANOVA—Dunnett, *p* < 0.001). **: Significant differences between groups (ANOVA—Dunnett, *p* < 0.0001).

Using the MTT assay, the B16F0 cell proliferation was quantified after 48 h of DETa treatment, and carboplatin (CBP) was used as a chemotherapeutic control (Figure 1a,b). The cells treated with DETa showed a notable cytotoxicity in a dose-response manner, as well as the cells treated with CBP. The potency of the effects was quantified by calculating the concentration of treatment needed to inhibit 50% of proliferation (EC_50_); in DETa-treated cells, the EC_50_ was 1395.58 ± 132 µg/mL, while in CBP-treated cells, the value reached 73.19 ± 1.6 µg/mL.

Then, to determine if the cytotoxicity observed was exerted by cytostatic or lethal effects, the number and viability of treated cells were quantified by the trypan blue dye exclusion assay (Figure 1c,d). After 48 h, the viability of DETa-treated cells decreased in a dose-response manner; at this time, lethality reached up to 29.38% in the 1320 µg/mL dose, and the calculated DL_50_ (treatment concentration where 50% of cell death was observed) was 1662.26 ± 35.2 µg/mL. On the other hand, the maximum dose of CBP used (150 µg/mL) only reduced cell viability by 13.79%, and the DL_50_ was estimated at values higher than 50 mg/mL.

Finally, the time-dependent effects of DETa were evaluated at 12, 24, 36 and 48 h after the initiation of treatment. The B16F0 cell proliferation was proportionally affected by the dose used (Figure 1e), and the DT (doubling time) was significantly increased from 18.2 h in control untreated cells to 27.9 h and 46.6 h in 880 or 1760 µg/mL DETa-treated cells, respectively (ANOVA—Dunnett, *p* ˂ 0.001). The cell viability was also reduced in relation to the duration of DETa treatment (Figure 1f). At 24 h, doses of 880 and 1760 µg/mL reduced the values from 96.41% in untreated controls to 94.87% and 51.45%, respectively. After 48 h, the values decreased from 97.61% in controls to 80.11% and 41.08%, respectively. Only the changes induced by DETa doses of 1760 µg/mL resulted in a significant reduction of viability compared to the values of controls (ANOVA—Dunnett, *p* < 0.001), confirming the DL_50_ calculation presented previously.

**Figure 2:**
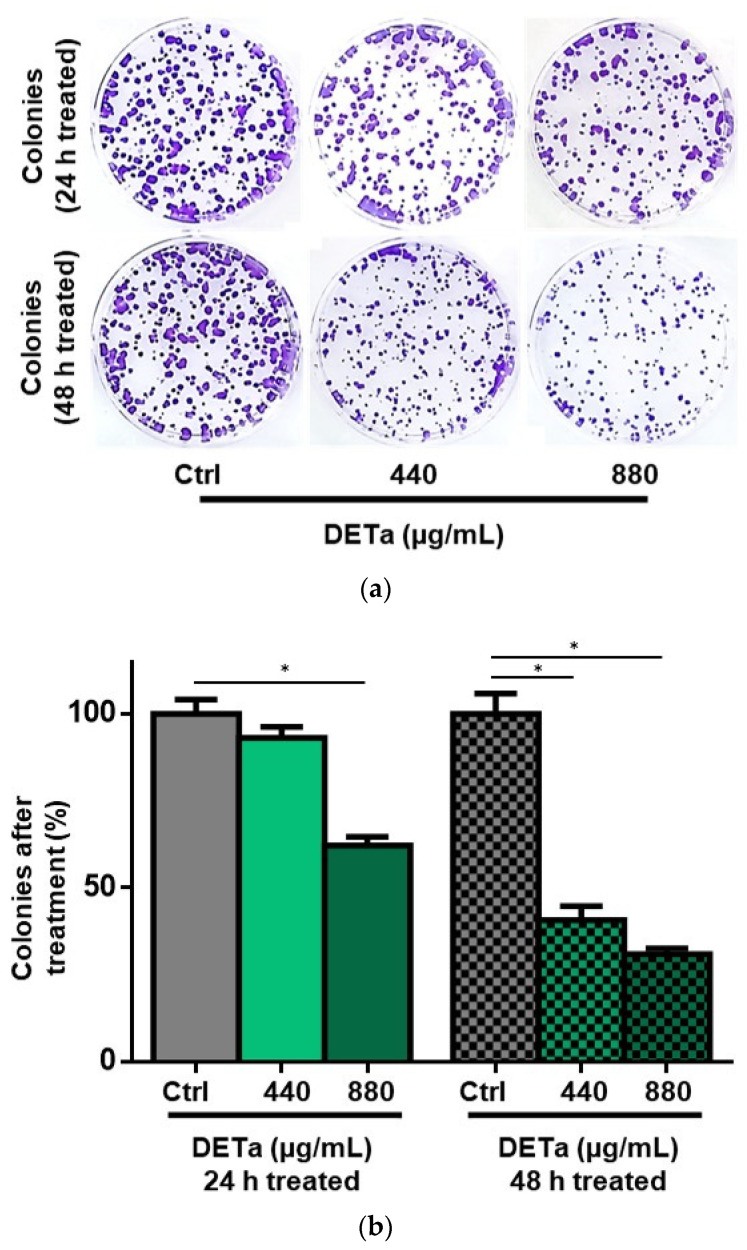
DETa diminishes clonogenicity of B16F0 melanoma cells. Panel (**a**) shows representative wells of positive colonies formed on the different treatment paradigms. Panel (**b**) presents the percentage of positive colonies formed after 24 and 48 h of treatment with 440 and 880 µg/mL DETa in relation to the control untreated cells. Ctrl: control untreated group. DETa: *T. absinthioides* decoction. CBP: Carboplatin. *: Indicates significant differences between groups (ANOVA—Dunnett, *p* < 0.0001).

Then, to evidence if DETa determines a long-term antiproliferative effect in treated cells, a clonogenic survival assay was performed. To analyze the cells’ capability to repopulate and form colonies after treatment, viable B16F0 cells were treated with 440 and 880 µg/mL DETa for 24 and 48 h. After treatment, cells were cultured in a fresh media without additional treatments for 7 days and positive colonies (defined as those containing at least 50 cells) were quantified (Figure 2a,b). Morphological details of positive and abortive colonies are shown in the Supplementary Figure S1.

The clonogenicity of 24 h treated cells was slightly reduced at the lower concentration of DETa used (440 µg/mL), resulting in a colony formation of 93.13% relative to control untreated cells, which were considered as 100%. However, the same DETa concentration applied for 48 h induced a significant reduction in the percentage of positive colonies formed, representing only 40.73% of the colonies formed by the untreated control cells (ANOVA—Dunnett, *p* < 0.0001). As expected from these results, the higher concentration used of DETa (880 µg/mL) evidenced a more pronounced reduction in clonogenicity. While the cells treated for 24 h reached 62.14% of positive colonies, the same concentration applied for 48 h reduced the positive colonies to 30.89%. In both cases, the resulting percentage of colonies was reduced significantly compared to the colonies formed by untreated control cells (ANOVA—Dunnett, *p* < 0.0001). When treatment duration was considered at the same doses (440 and 880 µg/mL), the percentage of positive colonies was significantly diminished at 48 h compared with the 24 h treatment (Student’s *t* test, *p* < 0.0001) in both cases.

Given the presented results, it is possible to affirm that DETa induces cytotoxicity on B16F0 non-metastatic melanoma cells, affecting cell proliferation, viability and clonogenicity in a dose and time-dependent manner.

### 2.2. In Vitro Study of DETa Effects on Melanoma Metastatic Cell Line Adhesion, Migration and Invasion

After establishing the cytotoxic effect of DETa in non-metastatic melanoma cells (B16F0), the sibling metastatic isogenic cell line (B16F10) was selected to analyze the effect of treatment on metastasis by determining its impact on the processes of adhesion, migration and invasion. To proceed with the study, non-antiproliferative doses were established for DETa and CBP after 24 h of treatment (See, Supplementary Figure S2). The maximum non-antiproliferative doses found in B16F10 at 24 h of treatment were 880 µg/mL for DETa and 20 µg/mL for CBP. These doses did not induce an increase in proliferation either. Consequently, both concentrations were selected to study the anti-metastatic effects of DETa *in vitro*.

**Figure 3:**
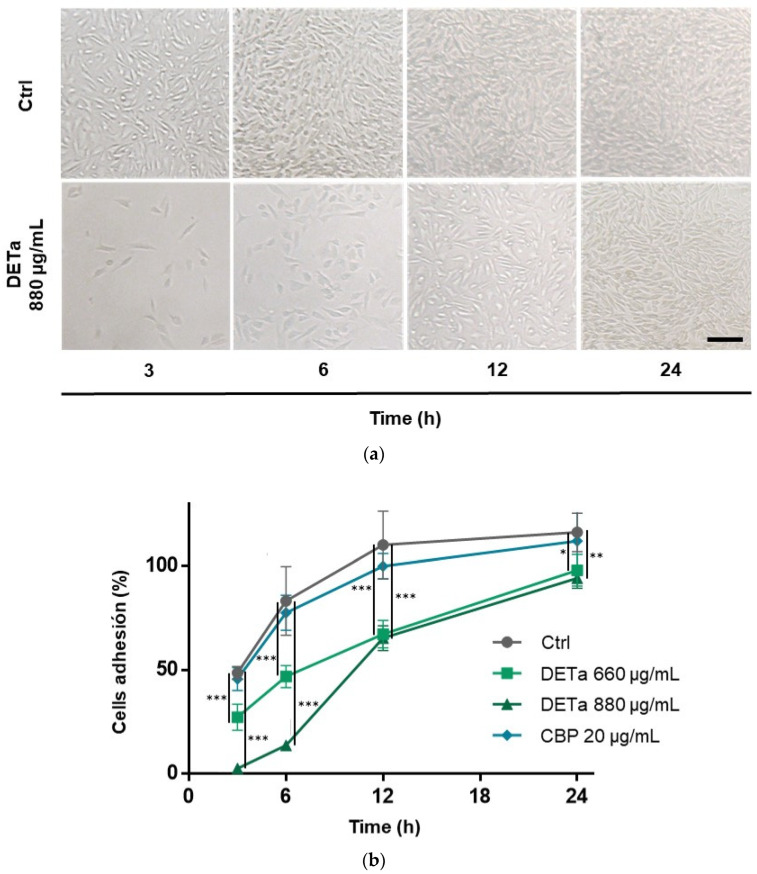
DETa decreases adhesion of B16F10 metastatic melanoma cells. Panel (**a)** shows a photographed comparison between the attached cells in the different treatment paradigms at 3, 6, 12 and 24 h. Bar: 150 µm. Panel (**b**) presents the percentage of attached cells at 3, 6, 12 and 24 h after plating when the seeding media contained DETa (660 or 880 µg/mL) and CBP (20 µg/mL) compared with untreated cells (Ctrl). Ctrl: control untreated group. DETa: *T. absinthioides* decoction. CBP: Carboplatin. *: Indicates significant differences between groups (ANOVA—Dunnett, *p* < 0.05). **: Significant differences between groups (ANOVA—Dunnett, *p* < 0.005). ***: Significant differences between groups (ANOVA—Dunnett, *p* < 0.0001).

Thereafter, to evaluate the effect of DETa on the adhesion capacity of the B16F10 cell line, after plating, the quantification of attached cells was performed at intervals of 3, 6, 12 and 24 h post-seeding (Figure 3a,b).

During the 24 h-analysis, at all intervals studied, cells treated with DETa exhibited a significant reduction in the percentage of attached cells compared to both CBP-treated and untreated cells (ANOVA—Dunnett, *p*< 0.05). Notably, DETa-treated cells evidenced diminution in attachment beginning at 3 h post-seeding. The dose of 880 µg/mL allowed 2.57 ± 1.7% of cells to attach, while the dose of 660 µg/mL allowed 27.22 ± 6.3%. In contrast, the percentage of attached untreated cells was 48.58 ± 3.2%. After 6 h, the maximum difference between DETa 880 µg/mL and the control cells was observed, with 13.70 ± 2.4% and 83.21 ± 6.5% of cells attached, respectively. Later, 12 h after cell plating, the maximum difference between DETa 660 µg/mL and untreated controls was observed (67.27 ± 5.6% and 110.1 ± 16.2%, respectively). After this time, differences between groups started to diminish, but they remained significant until the last measurement at 24 h, where the percentage of attached cells was 94.17 ± 4.9%; in 880 µg/mL DETa, 97.98 ± 7.6%; in 660 µg/mL DETa and 116.1 ± 9.3% in the untreated control cells. In contrast, CBP-treated cells only exhibited slight alterations in adhesion capacity when compared to the untreated cells at any time; in fact, this is consistent with the negative effect reported for CBP on melanoma cell line metastasis [18].

**Figure 4:**
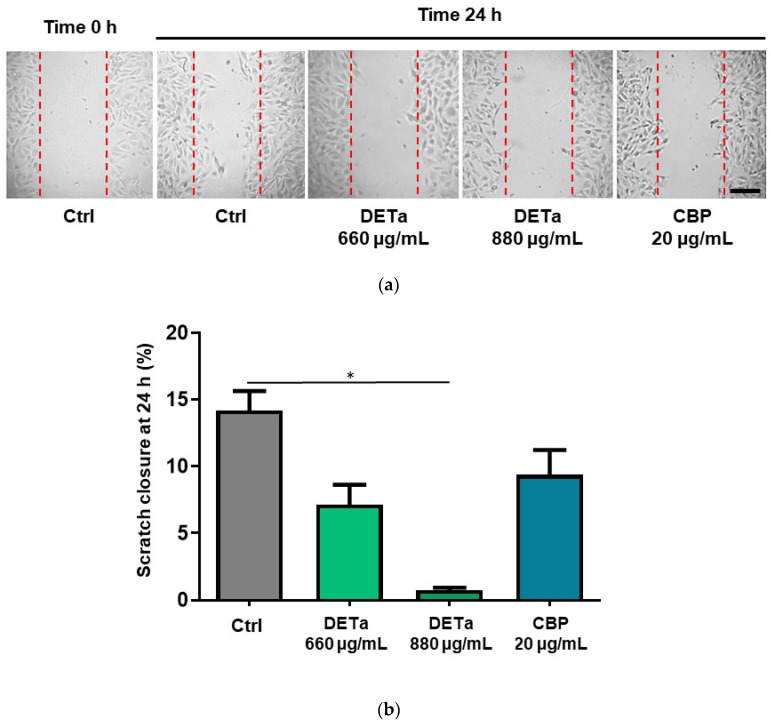
DETa reduces migration of B16F10 metastatic melanoma cells. Panel (**a**) shows the appearance of the scratch at time 0 and 24 h later, under the different treatment paradigms. Bar: 150 µm. Panel (**b**) exhibits the percentage of scratch closure after 24 h of treatment with 660 and 880 µg/mL DETa, and 20 µg/mL CBP in relation to the control untreated cells. Ctrl: control untreated group. DETa: *T. absinthioides* decoction. CBP: Carboplatin. *: Indicates significant differences between groups (ANOVA—Dunnett, *p* < 0.0001).

Then, to analyze if DETa treatment affects cell migration, a wound healing assay was performed (Figure 4a,b) using the same non-antiproliferative doses of treatment as those used in the adhesion assay (See Supplementary Figure S2).

After 24 h, all treatments reduced the scratch closure compared to the untreated control cells. However, the only treatment that resulted in a statistically significant reduction (ANOVA—Dunnett, *p* < 0.0001) was DETa 880 µg/mL, which markedly reduced the scratch closure by 0.76 ± 0.19%, while in the control untreated cells, the percentage of closure was 14.19 ± 1.4%. The other treatments, 660 µg/mL DETa and 20 µg/mL CBP, reduced the scratch width by 7.15 ± 1.5% and 9.38 ± 1.8%, respectively.

Finally, the study of cell invasion using the Boyden transwell chamber coated with Geltrex, after 24 h, did not demonstrate significant differences between the untreated control cells and those treated with DETa (660 and 880 µg/mL) or CBP (20 µg/mL). The obtained results are presented in Supplementary Figure S3.

Altogether, these findings suggest that DETa has *in vitro* antimetastatic activity, by disrupting the adhesion and migration processes in the B16F10 murine metastatic-melanoma cell line, without affecting the cell invasion capability.

### 2.3. Intestinal Permeability of DETa Phenolic Compounds

**Figure 5:**
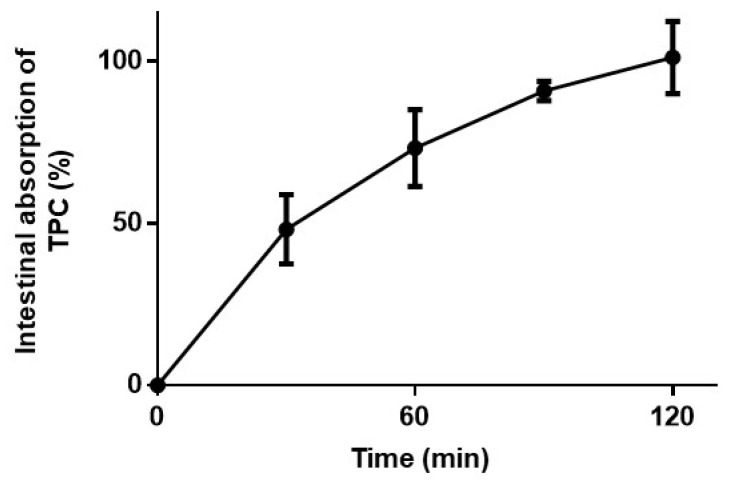
Kinetic absorption of TPC determined by the *ex vivo* intestinal sac, demonstrating the DETa phenolics bioavailability. TPC: Total Phenolic Content.

The bioavailability and bioaccesibility of active compounds are crucial determinants of their efficacy in performing biological functions, such as their antitumoral and antimetastatic activities. The non-everted intestinal sac model was employed to evaluate the intestinal absorption of the total phenolic content (TPC) present in DETa and its rate of absorption at 30, 60, 90 and 120 min (Figure 5). The observed absorption kinetics revealed a gradual increase, with 48.19 ± 10.6% of DETa TPC absorbed at 30 min, 72.29 ± 11.89% at 60 min and 90.91 ± 2.9% after 90 min. Notably, a complete absorption rate of 101.21 ± 11.2% was achieved at 120 min. This kinetic profile highlights the dynamic nature of DETa TPC absorption and its bioavailability, supporting the attribution of the systemic activity observed *in vivo* in melanoma tumors and their metastasis to DETa phenolic compounds.

### 2.4. In Vivo Demonstration of DETa Antitumor and Antimetastatic Effects

#### 2.4.1. Oral Administration of DETa Determines Antitumoral Effects on Induced Subcutaneous Melanoma

**Figure 6:**
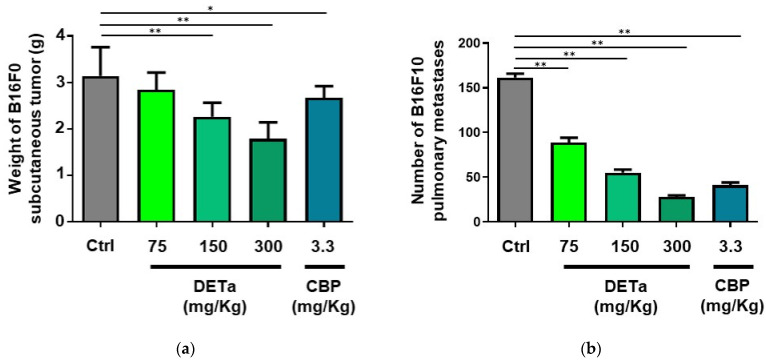
Antimelanoma systemic activity of DETa. Panel (**a**) presents the subcutaneous tumor weight induced by B16F0 cells after 28 days of treatment with DETa or CBP at the indicated doses. Panel (**b**) illustrates the number of total lung metastases induced by B16F10 cells tail injection and treated for 28 days with DETa or CBP at the indicated doses. Ctrl: control untreated group. DETa: *T. absinthioides* decoction. CBP: Carboplatin. *: Indicates significant differences between groups (ANOVA—Dunnett, *p* < 0.05). **: Significant differences between groups (ANOVA—Dunnett, *p* < 0.0001).

To assess the impact of orally administered DETa on tumor progression, male C57BL6/wt mice were subcutaneously inoculated with B16F0 cells and subsequently treated with 75, 150 and 300 mg/kg/day of DETa, *per os*, or 3.3 mg/kg/week of CBP, intraperitoneally (i.p.), as the chemotherapeutic positive control. An additional group, which did not receive any treatment, was used as a negative control. Following the experimental period of 28 days, animals were euthanized, and the tumors were dissected and weighed (Figure 6a). The subcutaneous tumor weight of the groups treated with DETa at 75, 150 and 300 mg/kg/day were 2.84 ± 0.37, 2.26 ± 0.31 and 1.79 ± 0.36 g, respectively. When the final tumor weight was statistically compared, the tumor reduction induced by DETa doses of 150 and 300 mg/kg/day were significantly diminished (ANOVA—Dunnett, *p* < 0.0001) compared to the untreated control group (3.14 ± 0.62 g). Additionally, a significant diminution in tumor weights was observed in the group treated with CBP (2.67 ± 0.25 g) when compared to the control group. These results emphasize the potential of DETa to interfere with melanoma tumor growth when acting systemically, demonstrating its antitumoral efficiency after oral administration.

#### 2.4.2. Oral Administration of DETa Reduces Pulmonary Metastasis of Melanoma

Afterward, to evaluate the effect of orally administered DETa on pulmonary metastasis of melanoma, male C57BL6/wt mice were injected with B16F10 cells into the lateral tail vein and then treated with DETa at 75, 150 and 300 mg/kg/day, *per os*, or CBP at 3.3 mg/kg/week, i.p. A fifth group of animals served as untreated control and did not receive any treatment. On day 28 after cell injection, animals were euthanized, lungs were dissected, and focal metastases were counted (Figure 6b). The DETa-treated groups exhibited a significant dose-dependent reduction in the number of focal pulmonary metastases when compared with the control group (ANOVA—Dunnett, *p* < 0.0001). While DETa treatment at 75 mg/kg/day reduced the metastasis count to 88.90 ± 17.10 (45% reduction), 150 and 300 mg/kg/day reduced the count to 55.1 ± 10.76 and 28.00 ± 5.77, respectively, resulting in a 66% and 82.7% reduction in total tumor numbers. Surprisingly, the CBP-treated group also demonstrated a notable reduction in lung metastasis, with a count of 41.3 ± 9.5. This outcome highlights the capability of DETa to interfere with the development of lung metastasis in melanoma when it is orally administered.

## 3. Discussion

In accordance with the *in vitro* obtained results on non-metastatic melanoma cells (B16F0), it is possible to affirm that DETa determines cytotoxicity by reducing cell proliferation and viability. Moreover, it affects long-term clonogenic survival, reducing the reproductive capacity of the cells to repopulate after treatment. Both cytotoxicity and clonogenicity were affected in a dose- and time-dependent manner. On their isogenic metastatic sibling cells (B16F10), DETa decreases the metastasis-related processes of adhesion, interfering with the capacity of cancer cells to attach to the culture surface and migration, reducing the cells’ ability to cover the wound-like surface. Using the *ex vivo* intestinal sac model, the absorption of DETa TPC was demonstrated, confirming the bioavailability of the phytochemicals present in the extract. Then, the systemic antitumoral and antimetastatic activities of orally administered DETa were demonstrated *in vivo*, determining a weight reduction of B16F0 subcutaneous induced tumors and decreasing the number of B16F10 pulmonary metastasis. Altogether, the presented evidence allows us to correlate the *in vitro* with the *in vivo* anticancer effects, positioning DETa as a botanical compound potentially useful for treating melanoma and its metastatic complications.

Since the aim of this study was to demonstrate the antitumoral and antimetastatic effects of DETa by correlating its *in vitro* and *in vivo* activities, no additional information was obtained to precisely demonstrate the specific mechanism or mechanisms of action involved. Despite this, considering the phytochemical composition of DETa, certain molecular pathways related to melanoma cell cytotoxicity and metastasis can be suggested.

In a previous study [14], our group analyzed the chemical composition of DETa, identifying sixteen compounds that serve as both bioactive and analytical markers: Apigenin, Caffeic acid, Caftaric acid, Chlorogenic acid, Ellagic acid, Epicatechin, Gallocatechin gallate, Ginnalin a, Myricetin, Naringin, OH-Tyrosol, *p*-Coumaric acid, Quercetin, Rosmarinic acid, Tessaric acid and Vanillic acid. To our knowledge, at least ten of these are phenolic compounds that have been studied in isolation for their beneficial activities against melanoma.

Among them, special attention should be given to the reports concerning Apigenin, Chlorogenic and *p*-Coumaric acids. These three phytochemicals were proposed among the cytotoxic markers of DETa by the chemical–biological correlation conducted on four different harvests of *T. absinthioides* plants [14]. In the case of Apigenin, it was demonstrated that this compound induces apoptosis in human melanoma cell lines (A375P and A375SM), upregulating the level expression of pro-apoptotic proteins Bax, p53, cleaved-PARP and cleaved-caspase 9. It was proposed that Apigenin induces apoptosis via regulating the Akt and MAPK signaling pathways. This capability to impair tumoral cell growth was also demonstrated *in vivo*, resulting in a decreased tumoral size in mice orally administered with Apigenin [19]. In addition, it was reported for its inhibitory effect on the migratory and invasive capacities of human melanoma A375 and G361 cell lines, as well as murine melanoma B16F10 cells. The antimetastatic effect was also demonstrated *in vivo* using the same mouse model used in our study, resulting in a number reduction of metastatic nodules in pulmonary tissue, suggesting that this compound inhibits the metastatic potential of B16F10 cells. This antimetastatic activity of Apigenin was related to the inhibition of the STAT3 signaling pathway, resulting in a decreased level expression of its target genes, such as MMP-2, MMP-9, VEGF and Twist-1, diminishing the epithelial-to-mesenchymal transition [20]. In relation to Chlorogenic acid, it has been reported as an antimelanoma compound based on evidence obtained from both *in vitro* and *in vivo* studies. *In vitro*, it reduced C32 melanoma cells’ viability, affecting the mRNA expression of the antioxidant enzymes superoxide dismutase-1 and glutathione peroxidase-1 [21]. Meanwhile, *in vivo*, Chlorogenic acid prevented the growth of B16F10-induced tumors by promoting the polarization of tumor-associated macrophages [22]. Additionally, *p*-Coumaric acid was described as an antimelanoma compound with the capability to inhibit proliferation and colony formation of human and murine melanoma cell lines (A375 and B16, respectively), showing similar effects to those described in this study for the murine cell line. The cellular mechanisms underlying these effects involved A375 cell cycle arrest at the S phase in A375 cells, mediated by downregulation of Cyclin-A and CDK2. In contrast, in B16 cells, G0-G1 phase arrest was observed, by the diminution of Cyclin-E and CDK2 expression. In both cell lines, *p*-Coumaric treatment led to apoptosis via the downregulation of Bcl-2 and the upregulation of Apaf-1, Bax, cytoplasmic cytochrome c, cleaved caspase-3 and cleaved caspase-9 [23].

The other phenolics compounds present in DETa, which have been previously reported for their anti-melanoma activities, are mentioned below. Caffeic acid induces G0/G1 cell cycle arrest, apoptosis and loss of reproductive capacity related to expression changes in Bax, Bcl-2 and caspases 1, 3 and 8 [24,25,26]. Ellagic acid decreases melanoma cells proliferation, viability, migration and invasion through modulation of the epidermal growth factor receptor pathway and suppression of the NF-κβ pathway [27,28]. In relation to catechins, they have been reported to inhibit proliferation, induce cell cycle arrest and promote apoptosis through activation of the mitochondrial signaling pathway [29]. Naringin has been reported to affect cell proliferation, increase apoptosis and inhibit migration and invasion processes as a consequence of the inactivation of the c-Src/Akt pathway [30]. Quercetin, in turn, was demonstrated to reduce melanoma cells migration through inhibition of FAK-paxillin-Akt expression [31] and the STAT3 signaling pathway [32]. To conclude this description of the mechanism of action of individual phytochemicals, Rosmarinic acid was reported to selectively inhibit the proliferation and viability of melanoma cells by disrupting the mitochondrial transmembrane potential [33].

These antimelanoma activities reported for the individual phenolic compounds present in DETa strongly support its effects on melanoma tumor growth and metastasis demonstrated in the present study; however, these findings consider only the effect of each isolated plant metabolite. In contrast, we propose that the combined presence of these metabolites within a complex phytochemical matrix leads to the compound interactions that underlie the properties observed in DETa. This intricate composition of bioactive compounds may act collectively, enhancing each other’s effects and contributing to the overall therapeutic potential. Since it is not possible to assign a specific molecular pathway affected by the phytochemical composition of DETA, it is important to consider the most widely accepted mechanisms of action reported for plant phenolic compounds in the modulation of cancer-related pathways. Accordingly, the most likely targeted intracellular processes include p53-regulated mechanisms related to cell cycle progression and apoptosis, the MAPK pathway involved in the regulation of cell growth, differentiation, survival and death and the PI3K/Akt/mTOR pathway, which controls functions such as cell proliferation, survival, growth and metastasis [5]. Despite this, the pharmacological interactions among DETa compounds, as well as their implications for their anti-melanoma actions, remain to be characterized, and future studies will be necessary to elucidate the cell-signaling cascades involved.

In the present study, we contribute to the current evidence on the potential benefits of including plant extracts or botanicals into melanoma therapeutic strategies. Regardless of the previously mentioned activities of the individual compounds, it is crucial to consider some reports about plant-derived extracts rich in phenolic compounds that have demonstrated activity against melanoma. There are some precedents that reliably confirm the efficacy of plant extracts to determine both the *in vitro* and *in vivo* effects regarding this particular type of cancer [34]. It has been reported that the phenolic compounds present in the extracts of *Crataegus azarolus* decrease cell viability and induce a significant reduction of weight and volume on B16F10 tumors when administered at doses of 150 mg/kg [35]. Similar antimelanoma activities were reported for the phenolic compounds present in *Daphne gnidium* aqueous extract, which showed antiproliferative activity against B16F10 melanoma cells and reduced tumor volume *in vivo* when administered at doses of 200 mg/kg [36]. Moreover, phenolic compounds derived from *Panax ginseng* decrease B16F10 *in vitro* invasion and migration, leading to a reduction in melanoma cell lung metastasis when orally administered at 1000 mg/kg in C57BL/6 mice [37]. In relation to these precedents, the results presented in this study are remarkable, as oral administration of DETa at doses starting from 150 mg/kg demonstrated significant antitumoral and antimetastatic effects. This concentration is achievable in 17 mL of the *T. absinthiodes* leaf’s aqueous extract. By extrapolating the doses and effects reported in this work, approximately 1000 mL would be required to achieve the same dose for an adult weighing 60 kg.

Overall, the bioavailability of DETa phenolic compounds after oral administration, along with its antimelanoma activities demonstrated in this study, represents a valuable contribution to the field of plant extracts as alternative and complementary options for cancer treatment. This work promotes further research on this botanical preparation, exploring its promising use in pharmaceutical or nutraceutical applications, thereby increasing the potential application of DETa beyond its traditional uses.

## 4. Materials and Methods

### 4.1. Plant Material and Aqueous Extract Preparation

*T. absinthioides* (Hook. & Arn.) DC. plants were collected in December 2022 in Mendoza, Argentina (32° 89′ 79.310″ S, 68° 87′ 57.630″ W), and the voucher specimen was deposited in the Mendoza Ruiz Leal herbarium (MERL 65309). DETa obtention and phytochemical composition was previously reported by our group [14], and the current preparation was controlled according to that study. Briefly, leaves were washed in water with sodium hypochlorite (1%, *v*/*v*). After being rinsed, they were dried until constant weight (2 weeks) in a 22–24 °C, ventilated chamber, protected from the sun’s radiation. Then, dry leaves were ground and stored until use at −20 °C. Decoctions were prepared by boiling 50 g of this plant material in 1 L of distilled water (5% p/v). After 10 min of boiling, the solid material was separated and the decoction was sterilized via filtration through a 0.22 µm pore size. DETa dry weight was calculated by placing 1 mL of the obtained decoction into a sterile Eppendorf at 37 °C using a cultivation incubator until the samples reached a constant weight. The obtained yields were 8.80 ± 0.02 mg/mL.

### 4.2. Cell Culture and In Vitro Treatment

The B16F0 and B16F10 murine melanoma cell lines (ATCC, VA, USA) were cultured in DMEM supplemented with 10% fetal bovine serum (FBS), penicillin (100 UI/mL), streptomycin (100 µg/mL) and 3.7 mg/mL of NaHCO_3_. All the cell culture reagents were purchased from Gibco (MA, USA). Cells were grown under a humidified atmosphere, at 37 °C and enriched with 5% CO_2_ (Panasonic CO_2_ Incubator MCO-19AIC, Osaka, Japan). When it is indicated, the treatments were added to the culture media 24 h after plating cells. Despite its low effectiveness in melanoma, CBP (Carboplatino®, Microsules, CABA, Argentina) was used as a positive control, as it is still considered a valid chemotherapeutic option [38,39,40]; however, CBP should not be used for dose comparison. Finally, proper volumes of sterile MilliQ water were used in the negative controls.

### 4.3. Cytotoxicity, Proliferation and Viability Determinations

To determine the treatment induced cytotoxicity, the MTT (3-(4,5-dimethylthiazol-2-yl)-2,5-diphenyl tetrazolium bromide) cell proliferation assay was used. Briefly, 3 × 10^3^/100 µL B16F0 and B16F10 cells were seeded in a 96-wells plate. After 24 h, the medium was replaced by fresh medium containing the treatments for the indicated period of time (24 or 48 h) until plate reading.

To quantify the cell number and viability, a trypan blue dye exclusion assay was performed in a dose-response and time-course experimental design. Briefly, 5 × 10^5^ B16F0 cells were plated into a 6-wells plate; 24 h later, media was replaced with fresh medium containing the treatments. After the indicated time, cells were trypsinized, incubated with trypan blue and quantified using a Neubauer hemocytometer chamber.

### 4.4. Clonogenic Survival Assay

To determine if treatments induce a long-term antiproliferative effect, the DETa-pretreated B16F0 cells, at 440 and 880 µg/mL doses, were used to perform a clonogenic survival assay. Briefly, 5 × 10^2^ untreated and post-treated viable cells were placed into a 6-well plate, in 2 mL of fresh media, without additional treatments, for 7 days. At this point, the remaining media was removed, and dishes were rinsed with phosphate buffered saline (PBS). Then, colonies were fixed with methanol and stained with crystal violet. Positive colonies, defined as groups of ≥50 cells, were scored with an inverted microscope (Zeiss 47 12 02—9901, Germany), considering the colonies formed by control cells as 100%. The resulting wells were photographed using a Nikon Coolpix S10 camera (Tokyo, Japan).

### 4.5. Cell Adhesion Assay

To assess whether treatments at non-antiproliferative doses interfere with cell attachment, 1 × 10^5^ B16F10 cells were seeded in a 6-well plate with culture media containing DETa at the indicated doses and CBP. To discard the potential influence of treatments’ cytotoxicity on the number of adhered cells, the maximum treatment doses used (880 µg/mL DETa and 20 µg/mL CBP) were previously determined as the maximum concentrations that did not affect cell proliferation in a prior MTT assay conducted for 24 h (See Supplementary Figure S2). At 3, 6, 12 and 24 h after plating, the culture media was aspirated, wells were rinsed twice with PBS and attached cells were trypsinized, incubated with trypan blue and quantified using a Neubauer chamber. Adhesion was calculated as the percent of cells counted in relation to the seeded cells at time 0 h (considered as 100%). Before wells were trypsinized, the attached cells were photographed using a Nikon Coolpix S10 camera.

### 4.6. Wound Healing Cell Migration Assay

The cell migration studies were performed via the wound healing assay. Briefly, 1 × 10^4^ B16F10 cells were seeded in a 96-well plate. After 24 h, confluent monolayers were wounded by pressing a sterile tip down onto the plate. Then, the medium was removed, wells were washed twice with sterile PBS and the medium containing treatments were added. Images at time 0 h were captured to record the initial width of the wounds. The cell migration towards the denuded area was reevaluated 24 h later. To avoid the interference induced by cell proliferation, the doses used were 880 µg/mL of DETa and 20 µg/mL of CBP, which did not induce proliferation changes after 24 h of treatment (See Suplementary Figure S2). Photographic control was performed using a Nikon Coolpix S10 camera in an inverted microscope, using the Image J 1.52a version software (NIH, MD, USA) and the polygon selection mode, determining the percentage of the wounded area at 24 h with respect to the control (0 h).

### 4.7. Cell Invasion Assay

The B16F10 melanoma cell invasion was assayed using a Boyden transwell chamber coated with Geltrex (Thermofisher Scientific, MA, USA) according to the manufacturer’s protocol. Briefly, transwell chambers with 24 wells of 8 µm pore size and coated with Geltrex were used. Cells at 1 × 10^5^/500 µL media without FBS and containing treatments were transferred to the upper chamber. As was mentioned previously, to avoid the interference induced by cell proliferation, the maximum doses used were 880 µg/mL of DETa and 20 µg/mL of CBP (See Supplementary Figure S2). The lower chamber was filled with fresh media, containing 10% FBS, to attract the cells. Chambers were then incubated for 24 h. At the end of the assay, inserts were removed, the non-invaded cells were wiped out with a cotton swab and the lower surfaces of the filter were fixed with methanol and stained with a 0.1% of crystal violet solution. In each transwell, the migrated cells were counted under a microscope at a magnification of 400× in 10 randomly selected fields.

### 4.8. Intestinal Absorption of Phenolic Compounds

The non-everted intestinal sac model is a simple and rapid technique performed to demonstrate the bioavailability of the TPC. The assay does not discriminate, considering different events such as passive and active diffusion, efflux and first-past metabolism. The current assay was modified from Ruan *et al*. [41]. Briefly, from CO_2_ euthanized C57BL6/wt animals (n = 3), 5 cm length of small intestines were obtained. The sacs were prepared and filled with 100 µL of DETa. Then, the non-everted sacs were incubated in 2 mL of oxygenated Tyrode buffer (136.9 mM NaCl, 2.7 mM KCl, 11.9 mM NaHCO, 4.2 mM NaH_2_PO_4_, 1.2 mM CaCl_2_·2 H_2_O, 0.5 mM MgCl_2_·6 H_2_O, 15 mM glucose) and incubated into an orbital shaker at 37 °C. The solution outside the sacs was sampled at 0, 30, 60, 90 and 120 min, restoring the final volume with fresh Tyrode buffer. The TPC was determined by the Folin–Ciocalteau method. The initial TPC of DETa was determined (161.39 µg/mL Gallic Acid Equivalents) and was considered to be 100%.

### 4.9. Animals and In Vivo Treatment

To determine whether DETa acts systemically to prevent tumor progression and metastasis, a murine melanoma model was performed in 6-week-old C57BL6/wt male mice. The animals were maintained under controlled light cycles (6:00 AM to 10:00 PM) and room temperature (22–24 °C). Drinking water and mice chow (GEPSA, Argentina) were available *ad libitum* and their consumption was quantified daily. The animals were used to perform two different assays: one related to the study of subcutaneous tumor progression, and another destined to study pulmonary metastasis. In each case, the mice were divided into five groups (n =10 each), according to the following description. One group was the untreated negative control (0 mg/kg); three groups were treated with DETa in a dose-response experimental paradigm, receiving 75, 150 and 300 mg/kg/day, *per os*, for 28 days; and finally, the positive control group, which received i.p. CBP (Carboplatino®, Microsules, CABA, Argentina) at 3.3 mg/kg/week. All animals were euthanized by CO_2_ asphyxia on day 28. 

To evaluate the tumor progression, the five groups of animals were separated and all animals were subcutaneously inoculated with 1 × 10^5^ B16F0 cells in the right flank (Day 1). After the animal’s euthanasia, the tumors were dissected and weighed. Additionally, to evaluate the pulmonary metastasis, another five groups of 10 animals each were conformed, and 1 × 10^5^ B16F10 cells were injected into the lateral tail vein (Day 1). After the animals were euthanized, the lungs were dissected, and focal metastases were counted under microscope.

All animals were cared for in accordance with the Guiding Principles in the Care and Use of Animals of the US National Institutes of Health. All procedures were approved by the Institutional Animal Care and Use Committee of the School of Medical Sciences, Universidad Nacional de Cuyo (Protocol approval N° 103/2017).

### 4.10. Statistics

*In vitro* experiments were performed three times in triplicate, the *ex vivo* intestinal sac used n = 3 models and the animal assays included n = 10 animals per group. All data are expressed as mean ± standard deviation (SD) and were analyzed using GraphPad Prism 8.0 software (Graph Pad Software, Inc., San Diego, CA, USA) to assign statistical differences. To assess EC_50_ and DL_50_, a sigmoidal dose-response analysis was performed. To determine the cell’s doubling time (DT), the exponential growth equation was used. For the EC_50_, DL_50_ and DT calculation, values were considered acceptable when goodness of fit resulted with R^2^ ≥ 0.95. For statistical comparisons, Student’s *t* test and one-way ANOVA followed by Dunnettt’s multiple comparison tests were used. Statistical significance was considered when *p* ˂ 0.05.

## 5. Conclusions

Based on the results obtained in this work, it is possible to confirm that DETa is bioavailable and exhibits systemic antimelanoma activity, reducing subcutaneous melanoma tumor growth and pulmonary metastasis when orally administered at doses starting from 150 mg/kg. While the antitumoral activity was correlated with the *in vitro* capability of DETa to diminish cell proliferation, viability and clonogenicity, the antimetastatic action was related to the reduction in the adhesion and migration capacity of melanoma cells. Although the aim of this study was strongly supported by the correlation between the *in vitro* and *in vivo* effects of DETa, the main limitation lies in the lack of precise elucidation of the mechanisms and pathways involved in its antimelanoma activity, as well as in the characterization of the pharmacological interactions among its phytochemical constituents. Altogether, the findings presented in this study position this botanical compound as a relevant agent for cancer research and treatment, promoting future studies to elucidate the molecular targets of DETa and explore its potential application to other cancer types.

## Data Availability

After publication, data will be placed at the “Repositorio de datos del CONICET” (https://ri.conicet.gov.ar/, accessed on 30 April, 2025).

## Supplementary Materials

The following supporting information can be downloaded at: https://www.mdpi.com/article/10.3390/plants14091379/s1, Figure S1: Morphological details of positive (Ctrl) and abortive colonies (DETa 440—880 µg/mL) obtained in the clonogenic survival assay. Figure S2: Study of DETa and CBP cytotoxicity in B16F10 metastatic melanoma cells. Figure S3: Boyden’s chamber transwell invasion assay results.
