# Supplementary material for: Tessaria absinthioides (Hook. & Arn.) DC. Determines Inhibition of Tumor Growth and Metastasis In Vitro and In Vivo in Murine Melanoma
Source: Plants (Basel). 2025 May 2;14(9):1379. doi: 10.3390/plants14091379 (PMC12073114; doi:10.3390/plants14091379)
Supplement: Supplementary file 1 [file plants-14-01379-s001.zip › Supplementary Figure S1.pdf]

Supplementary Figure S1:

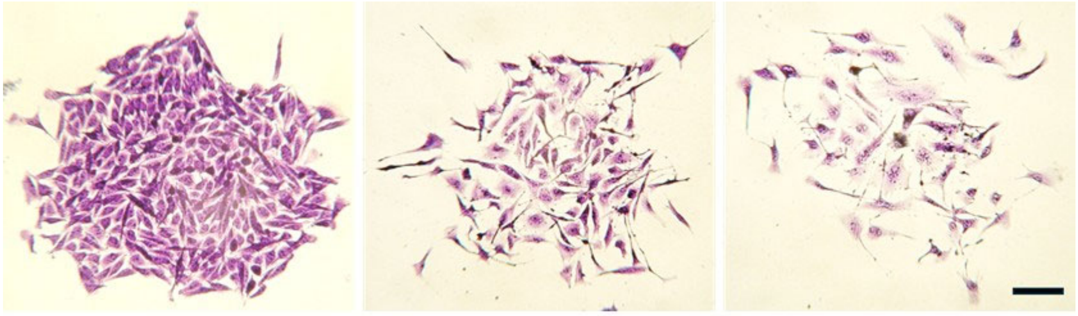

Supplementary Figure S1: Morphological details of positive (Ctrl) and abortive colonies (DETa 440 – 880 µg/mL) obtained in the clonogenic survival assay. While Ctrl colonies are evidenced for more than 50 cells and the normal B16F0 appearance, characterized by spindle-shape and epithelial-like resulted evident; in the previously DETa treated cells, abortive colonies are characterized by less than 50 cells morphologically altered. Bar indicate 150 µm. Ctrl: Control. DETa: *T. absinthioides* decoction.
