# Supplementary material for: Tessaria absinthioides (Hook. & Arn.) DC. Determines Inhibition of Tumor Growth and Metastasis In Vitro and In Vivo in Murine Melanoma
Source: Plants (Basel). 2025 May 2;14(9):1379. doi: 10.3390/plants14091379 (PMC12073114; doi:10.3390/plants14091379)
Supplement: Supplementary file 1 [file plants-14-01379-s001.zip › Supplementary Figure S2.pdf]

Supplementary Figure S2:

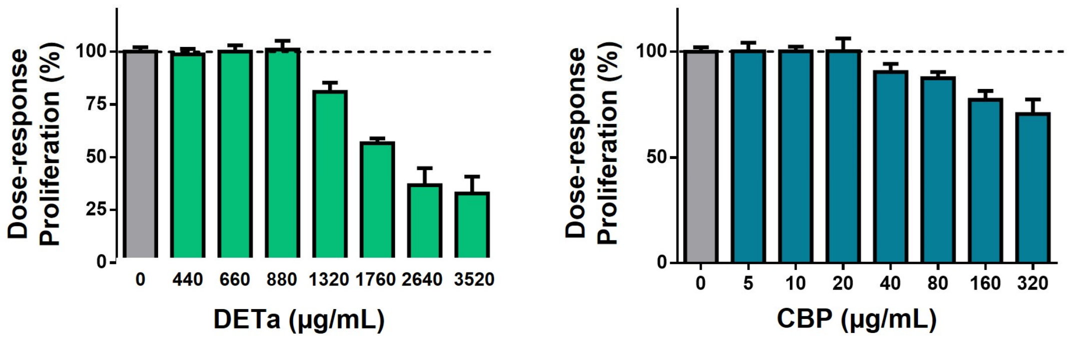

(**a**) (**b**)

Supplementary Figure S2: Study of DETa and CBP cytotoxicity in B16F10 metastatic melanoma cells. After 24 h of treatment, changes in proliferation of cultured cells were determined by MTT. Panel (**a**) exhibits a dose-response results after DETa treatment, where doses ⩽ 880 µg/mL did not affect proliferation. Panel (**b**) shows the results of CBP treated cells, where no affection of proliferation was observed at ⩽ 20 µg/mL. In consequence, DETa doses ⩽ 880 µg/mL and CBP ⩽ 20 µg/mL were selected for the *in vitro* antimetastatic studies. Ctrl: Control. DETa: *T. absinthioides* decoction. CBP: Carboplatin.
