# Supplementary material for: Tessaria absinthioides (Hook. & Arn.) DC. Determines Inhibition of Tumor Growth and Metastasis In Vitro and In Vivo in Murine Melanoma
Source: Plants (Basel). 2025 May 2;14(9):1379. doi: 10.3390/plants14091379 (PMC12073114; doi:10.3390/plants14091379)
Supplement: Supplementary file 1 [file plants-14-01379-s001.zip › Supplementary Figure S3.pdf]

Supplementary Figure S3:

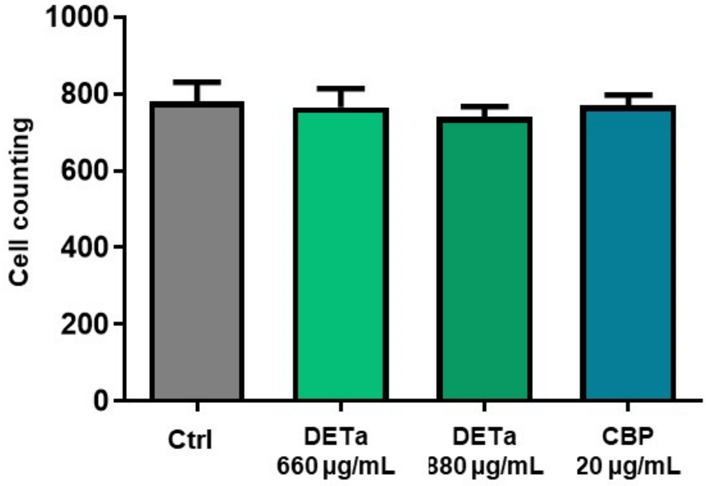

Supplementary Figure S3: B16F10 Boyden´s chamber transwell invasion assay results. Comparisons of the cell counts in different groups of B16F10 cells. Ctrl: Control. DETa: *T. absinthioides* decoction. CBP: Carboplatin.
